# High-pitch CT pulmonary angiography (CTPA) with ultra-low contrast medium volume for the detection of pulmonary embolism: a comparison with standard CTPA

**DOI:** 10.1007/s00330-023-10101-8

**Published:** 2023-09-01

**Authors:** Tobias Schönfeld, Patrick Seitz, Christian Krieghoff, Slavica Ponorac, Alexander Wötzel, Stefan Olthoff, Sebastian Schaudt, Jonas Steglich, Matthias Gutberlet, Robin F. Gohmann

**Affiliations:** 1 Department of Diagnostic and Interventional Radiology, Heart Center Leipzig, Strümpellstr. 39, 04289 Leipzig, Germany; 2 Department of General and Geriatric Medicine, St. Elisabeth-Hospital Leipzig, Biedermannstr. 84, 04277 Leipzig, Germany; 3 Medical Faculty, University of Leipzig, Liebigstr. 27, 04103 Leipzig, Germany; 4 Institute of Radiology, University Medical Centre Ljubljana, Zaloška Cesta 7, 1000 Ljubljana, Slovenia; 5 Emergency Department, Helios Park-Clinic Leipzig, Strümpellstr. 41, 04289 Leipzig, Germany; 6 Leipzig Heart Institute, Russenstr. 69a, 04289 Leipzig, Germany

**Keywords:** Pulmonary embolism, Computed tomography pulmonary angiography, Dual-source pulmonary angiography, High-pitch mode, Motion artifacts

## Abstract

**Objective:**

To investigate the feasibility and image quality of high-pitch CT pulmonary angiography (CTPA) with reduced iodine volume in normal weight patients.

**Methods:**

In total, 81 normal weight patients undergoing CTPA for suspected pulmonary arterial embolism were retrospectively included: 41 in high-pitch mode with 20 mL of contrast medium (CM); and 40 with normal pitch and 50 mL of CM. Subjective image quality was assessed and rated on a 3-point scale. For objective image quality, attenuation and noise values were measured in all pulmonary arteries from the trunk to segmental level. Contrast-to-noise ratio (CNR) was calculated. Radiation dose estimations were recorded.

**Results:**

There were no statistically significant differences in patient and scan demographics between high-pitch and standard CTPA. Subjective image quality was rated good to excellent in over 90% of all exams with no significant group differences (*p* = 0.32). Median contrast opacification was lower in high-pitch CTPA (283.18 [216.06–368.67] HU, 386.81 [320.57–526.12] HU; *p* = 0.0001). CNR reached a minimum of eight in all segmented arteries, but was lower in high-pitch CTPA (8.79 [5.82–12.42], 11.01 [9.19–17.90]; *p* = 0.005). Median effective dose of high-pitch CTPA was lower (1.04 [0.72–1.27] mSv/mGy·cm; 1.49 [1.07–2.05] mSv/mGy·cm; *p* < 0.0001).

**Conclusion:**

High-pitch CTPA using ultra-low contrast volume (20 mL) rendered diagnostic images for the detection of pulmonary arterial embolism in most instances. Compared to standard CTPA, the high-pitch CTPA exams with drastically reduced contrast medium volume had also concomitantly reduced radiation exposure. However, objective image quality of high-pitch CTPA was worse, though likely still within acceptable limits for confident diagnosis.

**Clinical relevance:**

This study provides valuable insights on the performance of a high-pitch dual-source CTPA protocol, offering potential benefits in reducing contrast medium and radiation dose while maintaining sufficient image quality for accurate diagnosis in patients suspected of pulmonary embolism.

**Key Points:**

*• High-pitch CT pulmonary angiography (CTPA) with ultra-low volume of contrast medium and reduced radiation dose renders diagnostic examinations with comparable subjective image quality to standard CTPA in most patients.*

*• Objective image quality of high-pitch CTPA is reduced compared to standard CTPA, but contrast opacification and contrast-to-noise ratio remain above diagnostic thresholds.*

*• Challenges of high-pitch CTPA may potentially be encountered in patients with severe heart failure or when performing a Valsalva maneuver during the examination.*

## Introduction

Pulmonary arterial embolism (PE) is the third most frequent acute cardiovascular disease (estimated annual incidence: 39–200 per 100,000 inhabitants), being second only to myocardial infarction and stroke [1, 2]. Rapid and accurate diagnosis of this potentially acutely or chronically life-threatening disease is crucial for the adequate management in order to improve patients’ prognosis [1, 3]. However, over-diagnosis and unnecessary treatment needs to be avoided [1, 3].

For several decades, computed tomography pulmonary angiography (CTPA) has been the method of choice for imaging the pulmonary vasculature in patients with suspected PE due to its high sensitivity (83%) and specificity (96%), availability, and short examination time [1, 4, 5]. Additionally, CTPA may render an alternative diagnosis, if PE is ruled out [1, 6].

It is well known that ionizing radiation may be harmful and 2% of all newly diagnosed neoplasia have been reported to be a result of radiation exposure from computed tomography (CT) [7]. Intravenous iodinated contrast medium may cause contrast-induced nephropathy [8–10] and has been reported to amplify DNA radiation damage during CT [11]. Despite the potential risks, CT imaging with iodinated contrast medium is indispensable for many CT applications, including vascular CT angiography and CTPA in particular.

So far, there have been numerous studies investigating scan protocols to optimize radiation dose or contrast medium volume without compromising image quality or diagnostic feasibility. For radiation dose, the most effective strategy has been to decrease tube potential [12–17], but also lowering tube current [18–22] and using the high-pitch mode in dual-source CT scanners have been explored. Scanning in high-pitch mode may reduce oversampling [23] and shortens image acquisition time [24]. Furthermore, image acquisition in high-pitch mode may mitigate or avoid motion artifacts without compromising image quality [25–28].

Motion artifacts frequently occur in patients with shortness of breath or chest pain, who have difficulty holding their breath [28]. Elderly individuals frequently have hearing or visual impairments that may also lead to a limited compliance during examination [29].

To mitigate the outlined challenges and risks during CTPA, some studies have combined more than one of these approaches (low tube voltage, high-pitch, low contrast medium volume) for the detection of PE [24, 30–32].

In this study, we investigated a CTPA protocol using ultra-fast high-pitch mode (pitch: 3.2), ultra-low contrast volume administration (20 mL), with kVp and tube current adaptation in normal weight, free-breathing patients for the detection of PE. The aim of the study was to explore the feasibility of this protocol in comparison to a standard CTPA in an emergency care setting.

## Material and methods

### Study design and patient selection

Our study was conducted in a single center. The study protocol complied with the Declaration of Helsinki (World Medical Association, version 2013) and was approved by the local ethics committee (registration number: 336/19-ek). Written informed consent was waived.

**Fig. 1:**
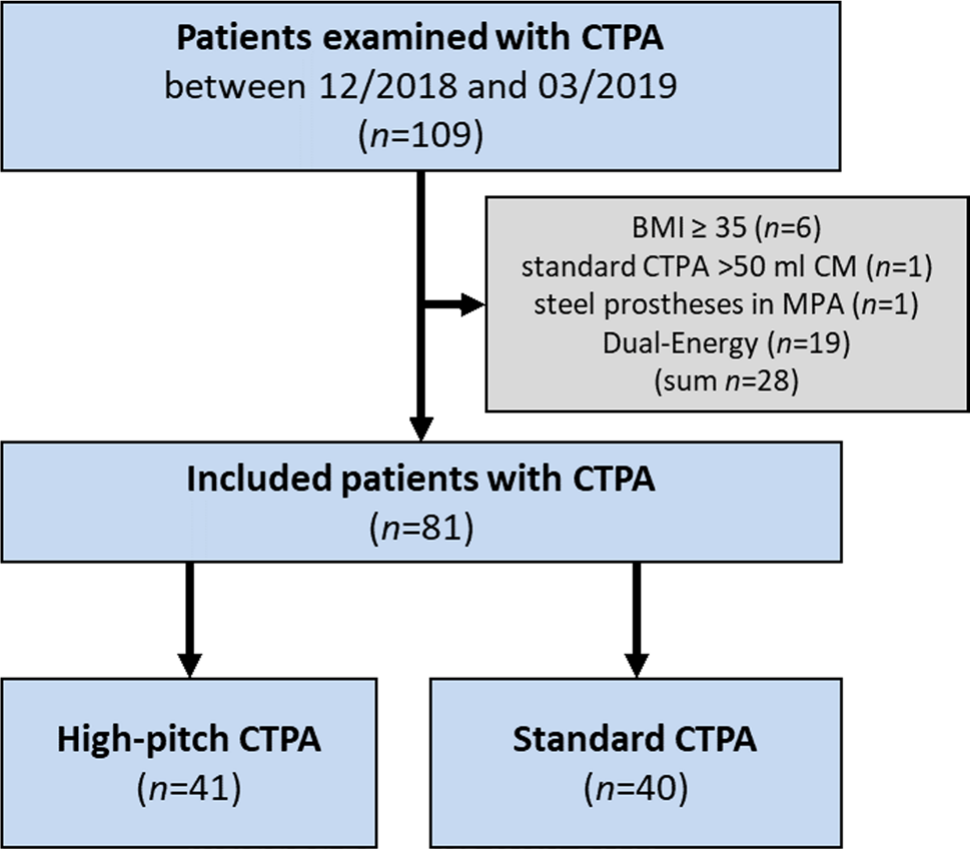
Flowchart of the study population. Flowchart of the study population according to the diagnostics received. BMI, body mass index; CTPA, computed tomography pulmonary angiography; CM, contrast medium; MPA, main pulmonary artery

Between December 2018 and March 2019, a total of 81 normal weight patients with a single contrast-enhanced scan of the thorax in pulmonary arterial phase were retrospectively included (Fig. 1). Of those, 41 patients have been examined with the high-pitch CTPA protocol and 40 patients with the standard CTPA protocol. All patients had been referred to our department with a clinical indication for CTPA because of suspected PE. Exclusion criteria were severe nephropathy (glomerular filtration rate < 30 mL/min), documented hypersensitivity to iodine-containing contrast media, obesity (BMI ≥ 35.0 kg/m^2^), or pregnancy. There was no selection or exclusion of possible medically or technically interfering conditions (e.g., critical illness, pacemaker, cardiovascular disease, or joint prostheses). Both protocols were used during the same time period; high-pitch CTPA was generally used during regular working hours; standard CTPA was generally preferred during night service hours.

### Image acquisition

All patients were examined with a second-generation dual-source CT scanner (SOMATOM Definition Flash, Siemens Healthineers). High-pitch CTPA was performed with dual-source single energy in helical scan mode (pitch: 3.2; scan time < 1 s); standard CTPA was performed with single source in standard helical scan mode (pitch: 1.2; scan time: ~ 2 s). Single-source CT scanners are generally not able to scan with a pitch beyond 1.5 without gaps in acquisition and resulting image artifacts; dual-source CT allows for scanning with a pitch beyond 3 without gaps in acquisition and artifacts [23]. For bolus tracking, the circular region of interest (ROI) (~ 1 cm^2^) was placed in the pulmonary trunk at the tracheal bifurcation in the scout-view for each protocol. The scan range (lung apices to costodiaphragmatic recess) for the both CTPA protocols was identical. Each scan was performed in craniocaudal direction with arms elevated above the head. For high-pitch CTPA, no breathing commands were given; for standard CTPA, patients were instructed to hold their breath during the scan (“hold your breath”). The contrast medium for both protocols was Iopromid (Ultravist 370, 370 mg Iodine/mL, Bayer Vital) applied via a peripheral venous catheter with a minimum size of 20 gauge at either upper limb. The contrast medium volume for high-pitch CTPA was 20 mL and for standard CTPA 50 mL, followed by a saline chaser. The rate of injection was 4.0 mL/s, respectively. For contrast medium administration, an automated contrast injector (Medrad Stellant, Bayer) was used. The scan was initiated 5 s after reaching a threshold of 80 HU (Hounsfield units) in the pulmonary trunk for both protocols. Further details of the protocols are given in Table 1.

**Table 1:** Scan protocols and image reconstruction

Parameter	High-pitch CTPA	Standard CTPA
Automatic tube voltage selection	Yes	Yes
Tube voltage range [kV]	70–100	70–120
Automatic tube current modulation	CARE Dose 4D*	CARE Dose 4D*
Tube current [ref. mAs]	200	100
Tube potential [ref. kV]	70	120
Pitch	3.2	1.2
Rotation time [s]	0.285	0.285
Table speed [mm/s]	431	161
Contrast medium volume [mL]	20	50
Contrast medium injection rate [mL/s]	4.0	4.0
Saline chaser volume [mL]	40	40
Saline chaser injection rate [mL/s]	4.0	4.0
Kernel	I31f	I31f
Section thickness [mm]	0.75	0.75
Increment [mm]	0.7	0.7
Iterative reconstruction algorithm	ADMIRE	ADMIRE
Strength of iteration	4	3

Technical parameters of applied protocols. *ADMIRE* Advanced Modeled Iterative Reconstruction (Siemens Healthineers)

### Image reconstruction

Images were reconstructed using the Advanced Modeled Iterative Reconstruction algorithm (ADMIRE, Siemens Healthineers) with a moderate strength of 4 for high-pitch CTPA and a strength of 3 for standard CTPA, respectively.

### Image quality assessment

#### Subjective image quality

Subjective image quality and vascular contrast opacification were assessed in the picture archiving and communication system (Sectra IDS7, version 23.2.0.5047, Sectra AB, version 23.2.0.5047) during the diagnosing process as a consensus by two radiologists with different levels of experience, one with 2–5 years and the other with a minimum of 7 years and rated on a 3-point scale (Table 2). All common tools for image settings were available. The quality rating score 1 denotes good to excellent image quality allowing detectability of PE down to subsegmental level, score 2 denotes adequate image quality with some artifacts or insufficient contrast opacification at subsegment level, and score 3 denotes examination with non-diagnostic image quality at segmental level.

**Table 2:** Subjective image quality

Quality rating score	High-pitch CTPA (*n* = 41)	Standard CTPA (*n* = 40)	*p* value
1	38 (93)	39 (98)	0.61
2	3 (7)	1 (3)	
3	0 (0)	0 (0)	
Total	1.07 ± 0.26	1.03 ± 0.16	

Quality rating score 1 is good to excellent; score 2 is adequate, with some artifacts, maybe non-diagnostic at subsegmental level; score 3 is non-diagnostic, repeated with additional contrast medium and/or optimized conditions. For group comparison, the chi-square test was used. Numbers are count and (percentage) or mean ± standard deviation. *CTPA* computed tomography pulmonary angiography

Information of detected PE was taken from the medical reports.

#### Objective image quality

To assess objective image quality, a validated free open-source software 3D Slicer (Version: Slicer 4.10.2, revision 28,257, built 2019–05-22, https://www.slicer.org/) was used [33].

Attenuation (quantified as mean CT number in HU) and noise (standard deviation [SD] of CT number in HU) were measured in each patient by placing a circular ROI in all pulmonary arteries from pulmonary trunk to segmental level (main pulmonary artery [*n* = 1], main pulmonary arteries [*n* = 2]; lobar arteries [*n* = 5]; segmental arteries [right: *n* = 10; left: *n* = 8]). The sizes of the ROI were adapted to the diameter of the artery to avoid interference with structures other than the contrasted blood. In the case of a clot or other pre-existing pathologies displacing the contrasted blood pool, the ROI was positioned proximally or distally to it or the corresponding vessel on the other side was used. The mean attenuation values of the paraspinal muscle were obtained by placing two ROIs into the muscle at the level of the pulmonary trunk. The background noise was measured by drawing four circular ROIs in the air (extracorporeal, ventral to the body at the level of main pulmonary artery). Segmentation was performed by a single observer with prior training.

The signal-to-noise ratio (SNR) and the contrast-to-noise ratio (CNR) were calculated as follows [15]:

$$SNR=\frac{mean\;attenuation\;value\left[HU\right]}{mean\;noise\;of\;air\left[SD\;of\;HU\right]}$$

$$CNR=\frac{mean\;attenuation\;value\left[HU\right]-attenuation\;value\;of\;erectorspinae\;muscle\left[HU\right]}{mean\;noise\;of\;air\left[SD\;of\;HU\right]}$$

### Measurements of radiation exposure

For radiation dose exposure estimation, we recorded the tube voltage (kV), dose-length product (DLP), and volume CT dose index (CTDI_vol_). Effective dose (ED) was calculated by multiplying the DLP with a chest-specific conversion factor for adults (0.014 mSv Gy^−1^ cm^−1^) according to international recommendations [34].

### Statistical analysis

Continuous variables are given as mean ± SD when normally distributed or as median and (interquartile range [IQR]) for non-normal distributions. Categorical and ordinal variables are given as count and (percentage). To check for normality of data distribution, histogram analysis was employed. For group comparison, an independent samples *t*-test was applied for continuous normally distributed variables, and the Mann–Whitney *U* test was used for categorical or non-normally distributed variables. The chi-square test was used to compare ordinal variables. A *p* value < 0.05 was considered statistically significant.

Data curation and documentation were performed with spreadsheets (Microsoft Excel Version 2019, Microsoft Corporation). For data analysis, statistical software (MedCalc Version 20.113, MedCalc Software Ltd.) was used.

## Results

### Patient/scan demographics and image findings

No statistically significant differences (*p* ≥ 0.09) in distribution of age, body mass index, sex, used tube potential, or detected PE between the two groups were found (Table 3).

**Table 3:** Patient/scan demographics and image findings

Parameter	High-pitch CTPA	Standard CTPA	*p* value
	*n* = 41	*n* = 40	
Age [years]	72.9 (64.0–81.8)	78.5 (69.1–81.9)	0.50
Weight [kg]	73.9 ± 17.5	80.2 ± 12.9	0.09
Height [m]	1.69 ± 0.10	1.71 ± 0.08	0.30
BMI [kg/m^2^]	25.5 ± 4.8	27.3 ± 4.0	0.09
Females	17 (41.5)	14 (35.0)	0.55
Tube potential [kV]			
70	2 (4.9)	0 (0)	0.63
80	12 (29.3)	18 (45.0)	
100	27 (65.9)	20 (50.0)	
120	0 (0)	2 (5.0)	
PE detected	8 (19.5)	9 (22.5)	0.74

Patient baseline characteristics at time of hospital stay. Numbers are mean ± standard deviation, median (interquartile range) or count and (percentage). *CTPA* computed tomography pulmonary angiography, *BMI* body mass index, *PE* pulmonary embolism

PE was identified on central, lobar, segmental, or subsegmental level in 3, 1, 3, and 1 patients in high-pitch CTPA and in 3, 2, 3, and 1 patients in standard CTPA, respectively.

### Image quality

#### Subjective image quality

The subjective image quality of both protocols was rated as good to excellent (rating score 1) in over 90% of all studies. There were no statistically significant differences between the protocols. The results of subjective image quality assessment are shown in detail in Table 2.

#### Objective image quality

High-pitch CTPA yielded significantly lower mean contrast opacification and noise values in all segmented pulmonary arteries compared to standard CTPA (283.18 [216.06–368.67] HU; 386.81 [320.57–26.12] HU; *p* = 0.0001). The attenuation in the images acquired with high-pitch CTPA was somewhat higher in the periphery, and lower centrally. Noise level and SNR in high-pitch CTPA were somewhat lower in the periphery, and higher centrally. CNR was higher in standard CTPA compared to high-pitch CTPA on every pulmonary level (*p* ≤ 0.01). The minimum CNR of any segmented artery in high-pitch CTPA was eight. Further details are shown in Table 4.

**Table 4:** Data of objective image quality for both protocols

Parameter/place of observation	ROI [*n*]	High-pitch CTPA	ROI [*n*]	Standard CTPA	*p* value
***Contrast opacification***					
Global [HU]	*1066*	*283.18 (216.06–368.67)*	*1040*	*386.81 (320.57–526.12)*	0.0001
MPA [HU]	41	293.10 ± 103.36	40	459.01 ± 148.77	< 0.0001
LPA [HU]	41	270.86 (220.48*–*368.31)	40	404.21 (326.91*–*532.44)	< 0.0001
RPA [HU]	41	277.25 (217.10*–*350.23)	40	420.05 (335.14*–*544.38)	< 0.0001
Lobar arteries right [HU]	123	282.31 (224.40*–*368.76)	120	406.66 (338.79*–*543.37)	< 0.0001
Lobar arteries left [HU]	82	279.53 (209.41*–*361.87)	80	390.55 (364.06*–*484.54)	< 0.0001
Segmental arteries right [HU]	410	294.37 (215.65*–*366.75)	400	384.18 (322.44*–*521.83)	0.0002
Segmental arteries left [HU]	328	291.05 (208.26*–*379.78)	320	372.36 (311.05*–*518.38)	0.0003
***Noise***					
Global [HU]	1066	28.77 ± 4.8	1040	34.05 ± 3.53	< 0.0001
MPA [HU]	41	31.51 ± 6.72	40	37.88 ± 4.42	< 0.0001
LPA [HU]	41	33.28 ± 6.89	40	38.29 ± 6.10	0.0009
RPA [HU]	41	33.15 ± 6.21	40	41.82 ± 7.07	< 0.0001
Lobar arteries right [HU]	123	30.4 ± 5.7	120	41.8 ± 8.5	< 0.0001
Lobar arteries left [HU]	82	32.76 ± 5.81	80	38.27 ± 4.04	< 0.0001
Segmental arteries right [HU]	410	27.41 ± 5.54	400	31.27 ± 4.54	0.001
Segmental arteries left [HU]	328	27.40 ± 4.91	320	31.58 ± 3.82	0.0001
***Image quality parameters***					
* SNR global*	*1066*	*10.47* ± *3.35*	1040	*12.54* ± *3.71*	0.01
SNR central	123	9.03 ± 2.66	120	11.28 ± 3.27	0.001
SNR lobar	205	9.47 ± 3.00	200	10.89 ± 3.07	0.03
SNR segmental	738	11.13 ± 3.77	720	13.47 ± 4.25	0.011
CNR global	*1066*	*8.79 (5.82–12.42)*	1040	*11.01 (9.19–17.90)*	0.005
CNR central	123	8.63 (5.89*–*11.19)	120	11.88 (10.0*–*17.34)	0.0004
CNR lobar	205	9.02 (5.60*–*12.37)	200	11.38 (9.46*–*17.8)	0.001
CNR segmental	738	8.83 (5.58*–*12.50)	720	11.06 (9.01*–*17.97)	0.014

The first ROI column corresponds to high-pitch CTPA and shows the number of segments used for respective statistical analysis, while the second ROI column correspond to standard CTPA. Variables are presented as mean ± standard deviation when normally distributed or as median (interquartile range) for non-normal distributions; mean values of contrast opacification and noise were calculated from all values of the segments listed above. *ROI* region of interests, *CTPA* computed tomography pulmonary angiography, *MPA* main pulmonary artery, *LPA* left pulmonary artery, *RPA* right pulmonary artery, *SNR* signal-to-noise ratio, *CNR* contrast-to-noise ratio

### Radiation dose measurements

The median ED of 1.04 (0.72–1.27) mSv/mGy·cm in our high-pitch CTPA group was significantly lower than that in the standard CTPA group with mean ED of 1.49 (1.07–2.05) mSv/mGy·cm, which resulted in about 30% of dose savings. An overview of DLP, CTDI_vol_, and ED is shown in Table 5.

**Table 5:** Estimations of radiation exposure

Parameter	High-pitch CTPA	Standard CTPA	*p* value
DLP [mGy·cm]	74.00 (51.55–90.63)	106.40 (76.60–146.65)	< 0.0001
CTDI_vol_ [mGy]	2.51 (1.91–2.95)	3.59 (2.72–5.07)	< 0.0001
ED [mSv/mGy·cm]	1.04 (0.72–1.27)	1.49 (1.07–2.05)	< 0.0001

Radiation dose is displayed as median (interquartile range). *CTPA* computed tomography pulmonary angiography, *DLP* dose-length product, *CTDI*_*vol*_ volume CT dose index, *ED* effective dose, calculated as DLP × conversion factor of thorax (*k* = 0.014) [34]

## Discussion

Image quality was diagnostic in high-pitch CTPA and standard CTPA in all cases with no difference in subjective image quality. However, objective image quality was somewhat lower in high-pitch CTPA compared to standard CTPA.

**Fig. 2:**
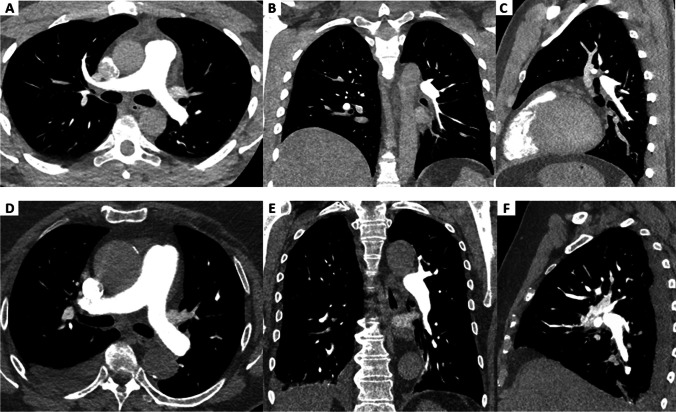
Comparison of the two protocols. Comparison of the two high-pitch CTPA (**A**–**C**) at 100 kVp of a 38-year-old male (BMI 28.7 kg/m^2^) and standard CTPA at 100 kVp of a 68-year-old female patient (BMI 30.1 kg/m.^2^) in axial (**A**, **D**), coronal (**B**, **E**), and sagittal (**C**, **F**) view. Subjective and objective image quality were excellent for both studies (mean opacification of the pulmonary arteries: 375.0 ± 27.2 HU; 441.3 ± 36.7 HU). Radiation dose for high-pitch CTPA was lower comparted to standard CTPA (DLP: 89.6 mGy·cm; 135.0 mGy·cm, CTDI_vol_: 3.2 mGy/cm; 4.9 mGy/cm and ED: 1.3 mSv/mGy·cm; 1.9 mSv/mGy·cm). BMI, body mass index; CTDI_vol_, volume CT dose index; CTPA, computed tomography pulmonary angiography; DLP, dose-length product; ED, effective dose, calculated as DLP × conversion factor of thorax (*k* = 0.014 [34])

Despite subjective image quality being not significantly different between the groups, objective image quality was lower in high-pitch CTPA. Regardless, contrast opacification and CNR were above 270 (220.48–368.31) HU and 8, respectively, in all measured segments. Thus, with values systematically above 250 HU, the minimum contrast opacification of the pulmonary arteries to detect clots and to allow sufficient differentiation from neighboring structures and tissues reliably was reached also with high-pitch CTPA [27, 35]. Moreover, a minimum CNR of only five has been reported to be required for reliable detection of pulmonary embolism [27]. This threshold was exceeded in all segmented pulmonary vessels. An example of the image quality of our high-pitch CTPA protocol compared to the standard protocol is shown in Fig. 2. Overall, the lower objective image quality of high-pitch CTPA with ultra-low contrast medium volume did not seem to have a relevant influence on diagnostic confidence.

**Fig. 3:**
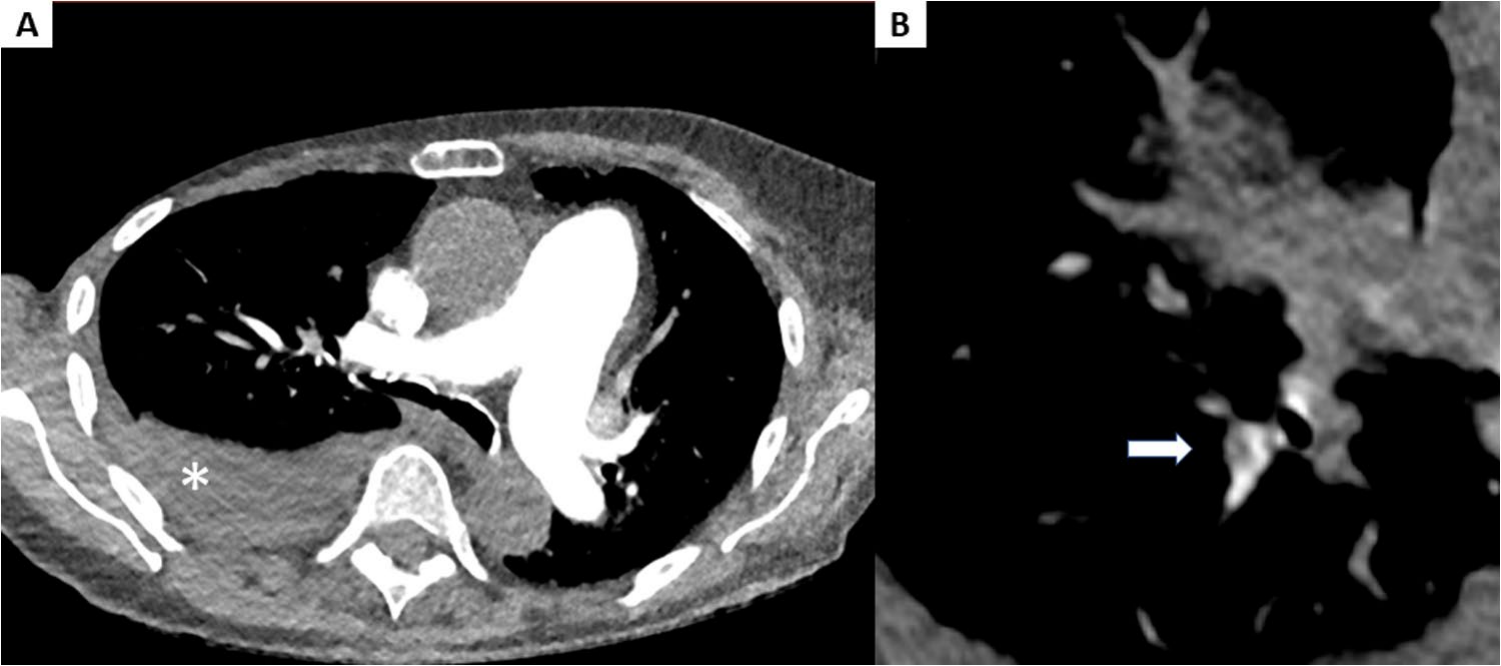
Representative example of a triple low examination. High-pitch CTPA of a 94-year-old female patient (BMI 25.8 kg/m^2^) at 70 kVp using 20 mL of contrast medium. Subjective and objective image quality were excellent (rating score = 1; mean opacification of the pulmonary arteries = 432.2 ± 34.5 HU) (**A**). Note the small peripheral pulmonary emboli in a segmental artery of the right lower lobe (arrow) (**B**). Furthermore, a serous pleural effusion (asterisk) and a prominent pulmonary trunk can be noted (**A**). CTPA, computed tomography pulmonary angiography; BMI, body mass index; HU, Hounsfield units

Recently, Cantarinha et al. reported that a free-breathing high-pitch (pitch 2.2) CTPA protocol with a fixed volume of contrast medium (35 mL) and kVp adaptation in dyspnoeic patients results in low-dose radiation exposure without hindrance by respiratory artifacts [30]. We found similar results by using an even higher pitch and smaller amount of contrast medium (Cantarinha et al.: 145 ± 73 mGy·cm; our results: 74.00, IQR 51.55–90.63 mGy·cm). Another study that used the high-pitch mode (pitch 3.0), 30 mL of contrast medium at 4 mL/s, BMI adjusted tube potential (80–120 kVp), and tube current (130–150 mAs) demonstrated sufficient diagnostic images down to subsegmental arteries with low radiation dose (ED: 2.3 ± 0.8 mSv) in oncology patients [32]. This study was conducted using a BMI-dependent protocol, which may be difficult to implement in an emergency room setting due to a lack of knowledge of biometric details in some cases. Furthermore, Rajiah et al. did not use 70 kVp as tube potential because, according to the authors, it might be inappropriate for diagnosing PE. In two of our examinations, we utilized a tube voltage of 70 kVp and were able to obtain good subjective and objective image quality (for an example, see Fig. 3). Bunch et al. reported higher contrast opacification in high-pitch CTPA compared to a standard CTPA using the same amount of contrast medium, though using a 3-s longer delay for high-pitch CTPA (9 s vs 6 s) [25].

The most common causes for indeterminate findings of PE in CTPA are motion artifacts, followed by poor contrast enhancement or transient interruption of contrast inflow into the right heart or poor contrast timing [35, 36]. Motion artifacts account for about half of the cases of misdiagnosis of pulmonary embolism [37]. High-pitch CTPA scanning generally produces fewer indeterminate examinations, even in free-breathing patients, compared to standard CTPA, especially regarding motion artifacts [25, 36]. Likely, because of the high pitch, we did not observe any motion or breathing artifacts impeding the diagnosis in our study group.

Hassan et al. showed that high-pitch CTPA with free-breathing patients yielded better image quality with less motion artifacts and reduced radiation dose [26], taking into consideration that the combined use of an adjusted pitch (pitch 2.0–3.0) and a variable amount of contrast medium (30–60 mL) with a fixed tube voltage (100 kV) makes it difficult to compare the conclusions of this study with our investigation. Overall, high-pitch CTPA approaches can reduce or even prevent artifacts related to breathing, cardiac motion, and Valsalva-related artifacts even in freely breathing patients [25–28, 30].

Recent studies investigated high-pitch CTPA in combination with decreased tube voltage or contrast medium volume [24, 30–32]. Lowering tube voltage can increase attenuation of enhanced vasculature while decreasing the applied radiation dose, as the mean effective energy is closer to the k-edge of iodine (33.2 keV) [27]. Furthermore, high-pitch acquisition mode decreases scan time. Both potentially allow for a reduction of the required amount of iodinated contrast medium [24, 31].

Moreover, some studies reported significant radiation dose reductions with high-pitch acquisition CTPA, possibly secondary to short acquisition time and reduced data overlap in addition to the abovementioned reasons [24, 25, 30–32]. Nevertheless, it remains unclear whether the dose savings are due to tube current reduction in response to tube potential reduction rather than high-pitch mode itself [27].

**Fig. 4:**
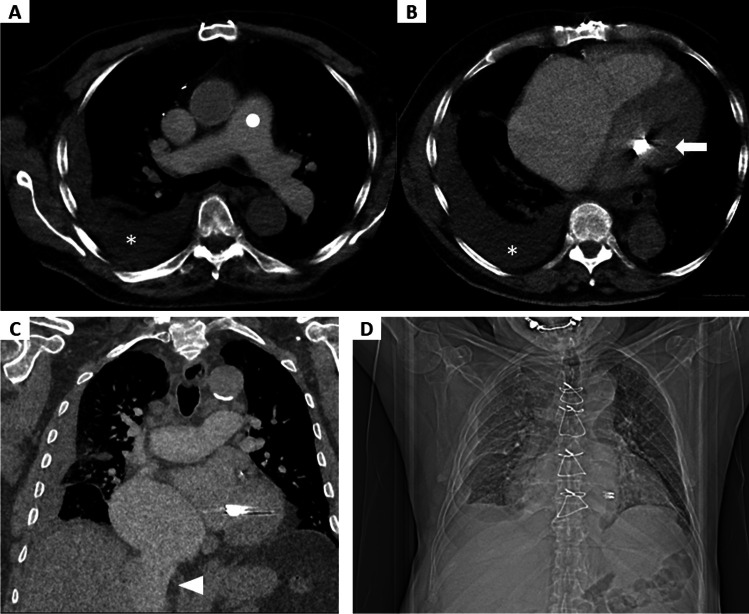
Example of an examination with adequate evaluated subjective image quality and several artifacts. Example of a high-pitch CTPA examination with adequate subjective image quality (rating score 2) without pulmonary arterial emboli in a 90-year-old male patient (BMI 24.9 kg/m^2^) (**A**–**C**). Scout-view of the same patient during the CT examination (**D**). The patient had severe heart co-morbidities, e.g., aortic valve replacement, 2 × mitral clipping because of mitral regurgitation, pulmonary hypertension with right-sided atrial dilatation and ride-sided pleural effusion (*) (**A**, **B**). Axial view with a circular region of interest (circle) and measured contrast opacification of 130.4 ± 28.4 HU (**A**). The arrow indicates radial beam-hardening caused by the mitral clips (**B**). Retrograde opacification of the inferior vena cava and hepatic veins (arrowhead) (**C**). The scout-view revealed signs of a severe heart disease. This should lead to a critical evaluation of the chosen protocol (**D**). BMI, body mass index; CTPA, computed tomography pulmonary angiography; HU Hounsfield units

In three high-pitch CTPA examinations, a retrograde opacification of the inferior vena cava and hepatic veins was observed due to right-sided heart failure and/or pulmonary hypertension. In two of these studies, diagnostic confidence was good (rating score 1); in one examination, the patient had severe heart co-morbidities (Fig. 4) and the subjective image quality was rated only as adequate (rating score 2). Thus, though bolus tracking was employed, severe heart disease may show a limitation of our proposed protocol.

Overall, our high-pitch CTPA protocol provided sufficient contrast opacification and CNR values for diagnostic images, which is eminent for the reliable diagnosis of pulmonary embolism. In addition, the radiation dose and the amount of iodinated contrast medium may be reduced by 30% and 60%, respectively, in comparison to the standard CTPA protocol. Furthermore, this high-pitch protocol can prevent the occurrence of motion artifacts secondary to shortness of breath or other cause of breathing incompliance because of the extremely short acquisition time. Retrograde contrast opacification may be observed without compromising diagnostic confidence by itself.

As the contrast opacification of the pulmonary arteries in our high-pitch CTPA with reduced volume of contrast medium volume was diagnostic but reduced compared to standard CTPA, other strategies, namely dual-energy CT, may be a more promising approach for the reduction of contrast medium volume in obese patients. However, the combination of high-pitch and dual-energy are only available in the latest generation of dual-source scanners with photon-counting detectors.

### Limitations

There are several limitations in this study and of our proposed protocol. First, this is a single center, retrospective design. The two radiologists were unblinded observers of subjective image quality and provided the results in consensus. Therefore, no conclusion on interobserver variability can be drawn and a certain bias may have occurred.

The CTPA images of both groups were reconstructed using the Advanced Modeled Iterative Reconstruction algorithm (ADMIRE, Siemens Healthineers) but with higher strength in high-pitch CTPA. This may be the reason for the lower noise levels in high-pitch CTPA despite the lower radiation dose applied in this group. However, as iterative reconstruction does not interfere with density levels, the effect likely is negligible and by no means explains the group differences [38].

Furthermore, in some examinations in our study group, we found poor contrast opacification in the pulmonary vasculature secondary to incomplete mixing of contrast medium with non-opacified blood as also described in previous investigations [27]. This may be of particular concern in less well ventilated and thus perfused lung areas, e.g., in case of consolidation, or in extremely well perfused areas, as potentially found in bronchopulmonary shunts, or in conditions with a combination of the two, e.g., in case of chronic PE or pulmonary hypertension. Therefore, an accurate delay time after contrast medium application is crucial for optimal opacification of the pulmonary arteries when using a small amount of contrast medium volume and high-pitch acquisition mode [24, 37]. It should be noted that using the same amount of contrast medium for each patient, regardless of their physical constitution and body habitus, resulted in a variation in enhancement of the pulmonary vasculature, as has also been reported previously [15].

Moreover, cardiac output has a considerable influence on vascular enhancement [37]. We did not evaluate these parameters, since it was not our focus. However, it may be advisable to screen patient’s heart function before referring them for CTPA, as it suggested to evaluate the severity also for other reasons, namely to stratify the mortality of PE (e.g., right ventricular overload or dysfunction) or to investigate alternative diagnosis, regardless [1].

However, the examination of a patient with severe heart diseases has shown artifacts that might represent an impairment of our proposed protocol. The sample size was too small to draw a conclusion. Thus, in such cases, it may be beneficial to use a larger volume of contrast medium or a standard CTPA protocol until other studies have further investigated the challenges of high-pitch CTPA in patients with severe cardiac comorbidities.

## Conclusion

Our high-pitch CTPA protocol using ultra-low contrast volume (20 mL) in normal weight patients rendered diagnostic images for the detection of pulmonary arterial embolism at concomitantly reduced radiation exposure compared to a standard CTPA. While subjective image quality was comparable, objective image quality was worse in high-pitch CTPA but likely still within accepted ranges for diagnosis. Therefore, careful patient preparation, precise contrast timing, and perhaps prior screening of the heart function may be advisable.

## Abbreviations

ADMIRE: Advanced Modeled Iterative Reconstruction
BMI: Body mass index
CNR: Contrast-to-noise ratio
CT: Computed tomography
CTDI_vol_: Volume CT dose index
CTPA: Computed tomography pulmonary angiography
DLP: Dose-length product
ED: Effective dose
HU: Hounsfield units
PACS: Picture Archiving and Communication System
PE: Pulmonary arterial embolism
ROI: Region of interest
SD: Standard deviation
SNR: Signal-to-noise ratio

## Declarations

### Guarantor

The scientific guarantor of this publication is Robin F. Gohmann, MD.

### Statistics and biometry

No complex statistical methods were necessary for this paper.

### Study subjects or cohorts overlap

No study subjects or cohorts have been previously reported.

### Methodology

*•* retrospective

*•* cross-sectional study

*•* performed at one institution
